# Consistent spectral predictors for dynamic causal models of steady-state responses

**DOI:** 10.1016/j.neuroimage.2011.01.012

**Published:** 2011-04-15

**Authors:** Rosalyn J. Moran, Klaas E. Stephan, Raymond J. Dolan, Karl J. Friston

**Affiliations:** a Wellcome Trust Centre for Neuroimaging, Institute of Neurology, University College London, 12 Queen Square, London WC1N 3BG, UK; b Laboratory for Social and Neural Systems Research, Institute for Empirical Research in Economics, University of Zurich, Zurich, Switzerland

**Keywords:** Mean-field models, Neural-mass models, Oscillations, Bifurcation, DCM, NMDA

## Abstract

Dynamic causal modelling (DCM) for steady-state responses (SSR) is a framework for inferring the mechanisms that underlie observed electrophysiological spectra, using biologically plausible generative models of neuronal dynamics. In this paper, we examine the dynamic repertoires of nonlinear conductance-based neural population models and propose a generative model of their power spectra. Our model comprises an ensemble of interconnected excitatory and inhibitory cells, where synaptic currents are mediated by fast, glutamatergic and GABAergic receptors and slower voltage-gated NMDA receptors. We explore two formulations of how hidden neuronal states (depolarisation and conductances) interact: through their mean and variance (mean-field model) or through their mean alone (neural-mass model). Both rest on a nonlinear Fokker–Planck description of population dynamics, which can exhibit bifurcations (phase transitions). We first characterise these phase transitions numerically: by varying critical model parameters, we elicit both fixed points and quasiperiodic dynamics that reproduce the spectral characteristics (~ 2–100 Hz) of real electrophysiological data. We then introduce a predictor of spectral activity using centre manifold theory and linear stability analysis. This predictor is based on sampling the system's Jacobian over the orbits of hidden neuronal states. This predictor behaves consistently and smoothly in the region of phase transitions, which permits the use of gradient descent methods for model inversion. We demonstrate this by inverting generative models (DCMs) of SSRs, using simulated data that entails phase transitions.

## Introduction

Magneto- and electro-encephalography (MEG, EEG) report the summed synaptic currents that flow in neural ensembles, where oscillations are a ubiquitous feature (Pfurtscheller and Lopes da Silva, 1999). Models of neuronal population activity causing these measurements have played a crucial role in studying the dynamic properties of cortex in healthy and pathological states (e.g. Stoffers et al., 2007; Breakspear et al., 2006; Robinson, 2006; Schnitzler and Gross, 2005; Liley and Bojak, 2005). These neuronal models can be combined with forward models, linking hidden neuronal states to experimental measurements. The resulting generative models can then be inverted (fitted to empirical data) using variational Bayesian techniques to make inferences about model parameters and the models per se (Friston et al., 2007). This is the basic idea underlying dynamic causal modelling (DCM) and has been implemented both for fMRI and electrophysiological data (e.g. David et al., 2005; Friston et al., 2003; Kiebel et al., 2006; Stephan et al., 2008). In particular, DCM for steady-state responses (SSR) (Moran et al., 2009) uses a biologically plausible generative model of neuronal population dynamics to explain cross-spectral densities from M/EEG or local field potential (LFP) recordings. Inverting these models provides *a posteriori* estimates of the model parameters that encode various synaptic properties, such as synaptic time-constants and connection strengths. DCM also provides an estimate of the model's evidence, allowing competing hypotheses about the underlying architecture to be tested.

Two broad classes of models have been used in DCM for electrophysiological data, namely, synaptic kernel convolution models (Marten et al., 2009; Valdes-Sosa et al., 2009; Wendling et al., 2000; Jansen and Rit, 1995; Lopes da Silva et al., 1976) and conductance-based models (Marreiros et al., 2009, 2010; Breakspear et al., 2003; Brunel and Wang, 2001). For both model classes, the activity of large neuronal populations is approximated by a probability density (see Deco et al., 2008 for review). In our previous exposition of DCM for SSR, we used a point mass or density to describe interactions of excitatory and inhibitory interneurons and pyramidal cell populations within a cortical source, a so-called neural-mass model (Deco et al., 2008; Moran et al., 2007, 2008). This model was of the kernel type, where postsynaptic responses result from the convolution of presynaptic input with a postsynaptic kernel. While this type of model offers a parsimonious description of population activity, conductance-based models are more directly related to specific synaptic processes. This is because they model different types of ionic currents explicitly, such as passive leak currents and active voltage and ligand-gated currents.

Marreiros et al. (2009) described a conductance-based model predicated on the Morris–Lecar (ML) model (Morris and Lecar, 1981). The ML model was extended to include three cell populations that are interconnected by means of fast glutamatergic and GABAergic synapses, forming a plausible cortical source. Given these sorts of models explain local field potential, electro-encephalographic and magneto-encephalographic data, they must necessarily accommodate thousands or millions of neurons. However, they have to generate (predict) the ensemble average that is actually measured. Fortuitously, under some simplifying (mean-field) assumptions, this average can be predicted quite simply. To do this we appeal to statistical mechanics and model the probability density over all hidden neuronal states (as opposed to modelling a large number of individual neurons and then taking the average over their states). This greatly finesses the numerics and allows us to model very (infinitely) large populations. Marreiros et al. (2009) characterised the ensemble density dynamics (that conform to the Fokker–Planck equation) using a Laplace approximation, where the voltages and conductances of the populations were summarised in terms of the sufficient statistics (mean and variance) of a Gaussian probability density function. Marreiros et al. (2009) compared a mean-field model (MFM) with dynamically coupled means and variances to a neural-mass model (NMM) where the variance was fixed. Importantly, they showed qualitative differences in the dynamic repertoire of the two systems with the MFM displaying limit-cycle attractors after bifurcation from a fixed-point. In this setting the mean-field model is inherently more nonlinear, because it entails nonlinear interactions between the mean and variance (sufficient statistics) of the hidden states. The emergence of limit cycles highlights an important limitation of most models used in DCM. To date, DCM has employed a global linearization (Valdes-Sosa et al., 2009) of nonlinear models, which assumes that changes in the (neuronal) states of the system can be approximated with small perturbations around a stable equilibrium point (Moran et al., 2007). This means that the model has a very limited dynamic repertoire, namely, a single fixed-point attractor. The work of Marreiros et al. and many others (Daffertshofer et al., 2000; Stam et al., 1997; Breakspear and Terry, 2002; Pijn et al., 1991; Babloyantz and Destexhe, 1988) has shown that nonlinearities can lead to a richer set of dynamic phenomena, with quasiperiodic, chaotic and itinerant attractors that can contribute to the spectral behaviour of electrophysiological data.

In this paper, we extend the physiological plausibility of the ML model described in Marreiros et al. (2009) by introducing NMDA ion channels that allow for slow synaptic currents in pyramidal cells and inhibitory interneurons (Brunel and Wang, 2001). This type of channel affords further nonlinearity to the system due to its voltage dependency (Jahr and Stevens, 1990), making the usual fixed-point approximation less appropriate. Here, we investigate the behaviour of this model over different parameter values and in different dynamic regimes. Put simply, we examine the frequency content of a neuronal system in two situations. One arises when the system settles to a fixed point (i.e., the average activity reaches steady-state). The spectrum observed is generated by noise, where the neurons act as a filter, shaping the noise spectrum to produce a profile of output frequencies. In a different dynamic regime, the average neuronal states themselves may oscillate. In this situation the system exhibits what is known as a quasiperiodic attractor and the frequency response to noise changes with different positions on the attractor. This means we have to take the average frequency response over the attractor manifold (i.e., over the limit cycle). Crucially, the frequencies that are preferentially filtered by the system are dominated by the frequency of the oscillation (limit cycle). This means the predicted spectral responses to noise under steady state can be seen (and treated mathematically) as a special case that obtains when the attractor collapses to a fixed point. In short, we predict spectral output from linearised perturbations at fixed-point attractors and over (limit-cycle) orbits on quasiperiodic attractors. This is the key contribution of this paper and affords an internally consistent method of spectral approximation for models that show bifurcations from fixed points to limit cycles and beyond. This is an important issue for nonlinear DCM, since changes in model parameters during inversion can cause bifurcations (phase transitions) and a qualitative change in attractor dynamics. These could induce phase transitions (discontinuities) in the objective function that is optimised during model inversion, which can easily confound the optimisation schemes used. It is this potential problem the current DCM for SSR was designed to finesse.

This paper comprises four sections. In the first, we review the conductance-based model, placing special emphasis on the new (NMDA) channel types introduced here. We present the model equations using the full mean-field treatment and relate them to their simplified (reduced) neural-mass form. In the second section, using numerical integration, we examine the effects of altering key parameters on the model's spectral behaviour, under noisy input. We apply the same treatment to mean-field and neural-mass forms and highlight the effects of coupling means and variances in the mean-field form. In the third section, we present a generic approach for generating (predicting) spectra that relies on sampling from the orbits of hidden neuronal states. Crucially, this spectral predictor is a smooth function of the parameters inducing bifurcations and furnishes a DCM of steady-state responses for nonlinear population dynamics that can exhibit phase transitions. We demonstrate this in the fourth section by inverting models of simulated spectra with different sorts of attractors. In doing this, we hope to demonstrate the face validity of model inversion, given ergodic behaviour, generated by qualitatively different dynamic mechanisms.

## Conductance-based neuronal ensemble model

The Morris–Lecar model (Morris and Lecar, 1981) was developed to explain a variety of oscillatory behaviours observed in barnacle muscle fibre, using a limited number of parameters. The model, originally comprising calcium and potassium channels, has been reorganised in this and previous accounts (Marreiros et al., 2010, 2009; Breakspear et al., 2003) to incorporate active neurotransmitter-gated synaptic processes. The kinetics encoding postsynaptic responses are formulated as an equivalent RC circuit where, using Kirchhoff's current law, capacitive synaptic current flow equals the summed active and passive currents across the membrane.

(1) $$C\dot{V}=\sum_{k}g_{k}(V_{k}-V)+u+\Gamma_{V}$$

Where *C* is the membrane capacitance set at 8 μF, *V* is the membrane potential, *g*_*k*_ is the conductance of channel *k*, *V*_*k*_ is its reversal potential, *u* is the applied input current and Г_*V*_ is the Gaussian (normal) noise of state *V*. In previous DCM applications (David et al., 2005; Stephan et al., 2007; Marreiros et al., 2009), we were primarily concerned with the action of active currents, that describe ligand-gated excitatory (Na^+^) and inhibitory (Cl^−^) ion flow, mediated through fast glutamatergic and GABAergic receptors such that

(2) $$C\dot{V}=g_{L}(V_{L}-V)+g_{E}(V_{E}-V)+g_{I}(V_{I}-V)+u+\Gamma_{V}$$

These sodium and chloride currents flow through the membrane with conductances *g*_*E*_ and *g*_*I*_ (described below) and reversal potentials of V_E_ = 60 mV and V_I_ = −90 mV, respectively. A potassium leak current is used to account for all passive ionic currents (Gutkin et al., 2003), with reversal potential V_L_ = −70 mV and conductance, g_*L*_, set to unity (Table 1). We also include a driving current input *u*(*t*) ∈ *R* to the granular (or stellate) layer. As above, the dynamics at the single-neuron level are stochastic, with *Γ*_*V*_ denoting Gaussian state noise (fluctuations) in voltage.

**Fig. 1:**
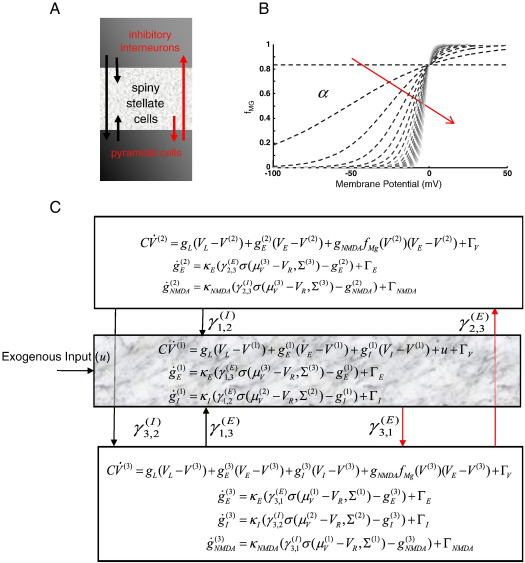
Source model. (A) Neuronal architecture for each cortical source comprising an input layer of spiny stellate cells, and supra/infra granular regions continuing inhibitory interneurons and pyramidal cells. Intrinsic connections between the subpopulations are drawn with arrows; red arrows indicate the presence of NMDA receptors, postsynaptically. (B) NMDA switch function (Eq. (3)) illustrated for increasing values of parameter α. As α increases, a voltage-dependent magnesium switch becomes highly nonlinear. (C) Stochastic equations describing the dynamical system with states comprising voltages and conductances.

In addition to the channels described above, we now include a third ligand-gated ion channel to model channels controlled by the NMDA receptor. NMDA receptor controlled ion channels (referred to as “NMDA channels” in the following) are special in that they are both ligand- and voltage-gated (Unwin, 1993). For an NMDA channel to open, following the binding of glutamate, there must first be a large transmembrane potential to remove a magnesium ion (*MG*) blocking the channel. Hence the dynamics for this particular current are given by an extended equation, which includes the magnesium component using the function *m*(*V*) (Tanaka, 2002; Wang, 2002; Durstewitz et al., 2000) with switch parameter *α*= 0.062 and reversal potential *V*_NMDA_ = 60 mV (Fig. 1B)

(3) $$\begin{array}{c} C\dot{V}=\sum_{k\in L,E,I}g_{k}(V_{k}-V)+g_{\text{NMDA}}m(V)(V_{\text{NMDA}}-V)+u+\Gamma_{V}\\ m(V)=\frac{1}{1+0.2\exp(-\alpha_{\text{NMDA}}V)} \end{array}$$

The temporal characteristics of all receptors in our model, either fast (e.g. AMPA, GABA_A_) or slow (NMDA), are incorporated in the equations of motion describing conductance (Table 1). These conductances *g*, which are dynamic states that depend on the presynaptic input *ς,* the number of open channels and the channel time-constants (1/*κ*), set to 4, 16 and 100 ms for AMPA, GABA_A_ and NMDA channels, respectively. These states are also subject to random fluctuations Г_*g*_

(4) $${\dot{g}}_{k}=\kappa_{k}(\varsigma_{k}-g_{k})+\Gamma_{g}$$

Here, *k* ∈ *E*, *I*, NMDA. Together, these stochastic differential equations describe the dynamics $\dot{x}=f(x,u)+\Gamma$ of the model's states $x=\{V,g_{L},\ldots ,g_{\mathrm{NMDA}}\}$ for a single neuron.

As established in Marreiros et al. (2009), we can transform these single-neuron stochastic dynamics to a deterministic generative model of ensemble or population dynamics using the Fokker–Planck formalism. A DCM of a single source typically comprises three coupled populations (David et al., 2005). Here, we employ a similar structure with interacting populations of excitatory spiny stellate cells, pyramidal cells and inhibitory interneurons, coupled through intrinsic connections (Fig. 1A and C). These populations contain the ion channels that mediate synaptic processing, with fast glutamate and GABA receptors in all populations and NMDA receptors in pyramidal and inhibitory populations (Hestrin et al., 1990; Homayoun and Moghaddam, 2007) (Fig. 1A and C).

The stochastic nature of the differential equations above is dealt with by decomposition into deterministic flow and diffusion using the Fokker–Planck equation, where the ensemble dynamics are described by$\dot{q}=-\nabla \cdot fq+\nabla \cdot D\nabla q$. Here, *q*(*x*) is the ensemble density over all hidden states $x=\{V,g_{L},\ldots ,g_{\mathrm{NMDA}}\}$ and the flow *f*(*x*, *u*) is the deterministic part of the motion of each state (Eqs. (3) and (4)), while the amplitude (covariance) of the random fluctuations is specified by the diffusion tensor *D*. The strength of this approach is that the Fokker–Planck formalism yields density dynamics of a deterministic form, even when the dynamics of each neuron are stochastic, nonlinear or even chaotic (Frank, 2004). A Laplace approximation further simplifies these equations (see Marreiros et al., 2009 for details), by assuming a Gaussian form for the population densities N(μ,∑). This is formally the same as approaches using the method of moments (Rodriguez and Tuckwell, 1998). Generally, for tractability, one only considers the first two moments. A Laplace approximation is motivated by the fact that a Gaussian density has the highest entropy (allows for the most uncertainty), given just two moments. The dynamics of the interactions among the sufficient statistics $\mu^{\left(j\right)}=\{\mu_{V}^{\left(j\right)},\mu_{L}^{\left(j\right)},\ldots ,\mu_{\mathrm{NMDA}}^{\left(j\right)}\}$ and *Σ*^(*j*)^ of each ensemble *j* ∈ 1, …, *n* (Fig. 1C) can then be written as an ordinary differential equation for all the sufficient statistics *μ* = {*μ*^(1)^, …, *Σ*^(1)^, …}

(5) μ˙=f(μ,u)μ˙i(j)Σ˙(j)=fi(j)(μ,u)+12tr(Σ(j)∂xxfi(j))∂xf(j)Σ(j)+Σ(j)∂xf(j)T+D(j)+D(j)T

where, ∂ _*x*_*f*^*j*^ and ∂ _*xx*_*f*^*j*^ (the gradient and curvature of the neuronal equations of motion) are non-zero within ensemble *j* (and ∂ *f*^(*j*)^/∂ *x*^(*i*)^ = 0 : ∀ *i* ≠ *j*; see Appendix A). These equations describe a mean-field model (MFM), in which the first and second order sufficient statistics interact, influencing each other when the curvature is non-zero.

In a system of connected neuronal ensembles, the input to one ensemble, *i*, now represents the expected firing rate from the source ensemble, *j* mediated through a coupling parameter for each channel type, *k*, *γ*_*ij*_^(*k*)^ : *k* ∈ *E*, *I*, NMDA (Fig. 1C)

(6) $$\varsigma_{k}^{}=\gamma_{i,j}^{\left(k\right)}\sigma (\mu_{V}^{\left(j\right)}-V_{R},\sum^{\left(j\right)})$$

Where *γ*_3, 1_^*E*^ = *γ*_3, 1_^NMDA^ = 0.5 are the coupling parameters linking the stellate population to pyramidal cells; *γ*_1, 3_^*E*^ = 0.5 couples pyramidal to stellate cells; *γ*_2, 3_^*E*^ = *γ*_2, 3_^NMDA^ = 1 couples pyramidal to inhibitory cells; *γ*_3, 2_^*I*^ = 1 couples inhibitory and pyramidal cells and *γ*_1, 2_^*I*^ = 0.25 is the coupling from the inhibitory interneurons to stellate cells. The sigmoid function *σ*(⋅), represents the cumulative distribution function (CDF) of the presynaptic depolarization density encoded by $N(\mu^{\left(j\right)},\sum^{\left(j\right)})$ around a threshold potential *V*_*R*_ = -40 mV which determines the proportion of afferent cells firing (see Marreiros et al., 2008). This function saturates at high firing rates. Its sigmoid form comes from mean-field assumptions and can be thought of as an average of an ensemble of (shifted) Heaviside or step functions modelling the all or nothing responses of individual neurons. The contribution from each cell is shifted according to its depolarisation. The slope of the sigmoid reflects the variance of this depolarisation over the population. In full mean-field treatments this variance changes as a function of time (see the Appendix). Simper (neural-mass) models assume this variance is constant and consequently the sigmoid has a fixed shape. See Marreiros et al. (2008) for a more detailed discussion.

Crucially, for our purposes Eq. (6) means that the flow depends on the population density. This is of fundamental importance: Without going into technical details, this means that the implicit Fokker–Planck equation (approximated to second order in Eq. (5)) becomes nonlinear. In turn, this means the density dynamics themselves can show quasiperiodic or chaotic behaviour as the collective behaviour of each population self-organises. This is a form of circular causality, in which the mean-field effect (the average state over one population) affects the activity of all neurons in another population, which determines its mean-field. See Breakspear et al. (2010) for a very nice introduction to nonlinear Fokker–Planck dynamics in the context of the Kuramoto model. In the absence of this coupling, any oscillatory behaviour of single neurons would desynchronise over time, because of the random effects and the population density would asymptote to a fixed-point. However, with coupling, richer dynamics can emerge. It is the emergence of periodic behaviour in the population dynamics we want to accommodate in our DCM.

This mean-field model can be simplified to form a neural-mass model (NMM). For the NMM, we use a static covariance. This results in equations of motion for the population voltage that comprise the flow term and a decay term.

(7) $${\dot{\mu }}_{V}^{\left(j\right)}=f_{V}^{\left(j\right)}(\mu ,u)+\frac{1}{2}tr(\sum^{\left(j\right)}\partial_{\mathrm{xx}}f_{V}{}^{\left(j\right)})$$

For the population conductance, a zero curvature means that these dynamics are solely determined by the flow.

(8) $${\dot{\mu }}_{k}^{\left(j\right)}=f_{k}^{\left(j\right)}(\mu ,I)$$

Although Eq. (8) can, in principle, generate limit cycles, the numerical simulations in Marreiros et al. (2009) suggest that fixed-point attractors predominate in the absence of NMDA channels. This contrasts with the mean-field formulation in Eq. (6), which is sufficiently nonlinear to generate limit cycles, even without (nonlinear) NMDA channels. This completes the description of the dynamics of the mean-field and neural-mass models used in subsequent sections. In what follows, we will simulate spectral responses using both the MFM and NMM models of one cortical region.

## Bifurcation analysis

**Fig. 2:**
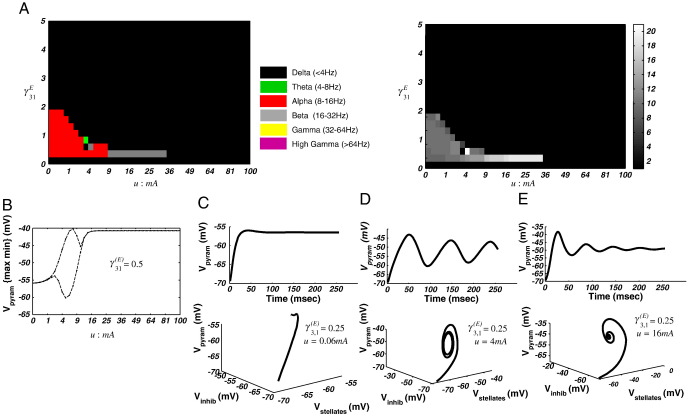
Spectral response of the neural-mass model. (A) Left: Spectral phase-diagram illustrating the maximal frequency band over the *u* and *γ*_31_^*E*^ dimensions. Right: phase-diagram illustrating the maximum frequency with 1 Hz resolution, which illustrates the highest maximum frequency of 21 Hz. Note that in these grey scale images, one can observe changes in frequency within a particular band, where here at *γ*_31_^*E*^ = 0.25, for example, we see an increasing frequency in the alpha limit cycle regime before it returns to a beta fixed point. (B) Bifurcation diagram illustrating the maximum and minimum pyramidal cell membrane potential along parameter *u* for *γ*_31_^*E*^ = 0.5. At *u* ~ 3.06 mA the system bifurcates and enters a limit-cycle attractor. Then at *u* ~ 10.6 mA, the system undergoes a second phase transition back to a fixed-point. (C) Time domain and phase domain portraits for one region of the 2-D parameter plane (*u* = 0.06 mA; *γ*_31_^*E*^ = 0.25), where the system lies at an alpha fixed-point. (D) Time domain and phase domain portraits for one region of the 2-D parameter plane (*u* = 4 mA; *γ*_31_^*E*^ = 0.5), where the system enters an alpha limit cycle. (E) A second region in parameter space, here at a beta fixed-point, when (*u* = 16 mA; *γ*_31_^*E*^ = 0.25).

**Fig. 3:**
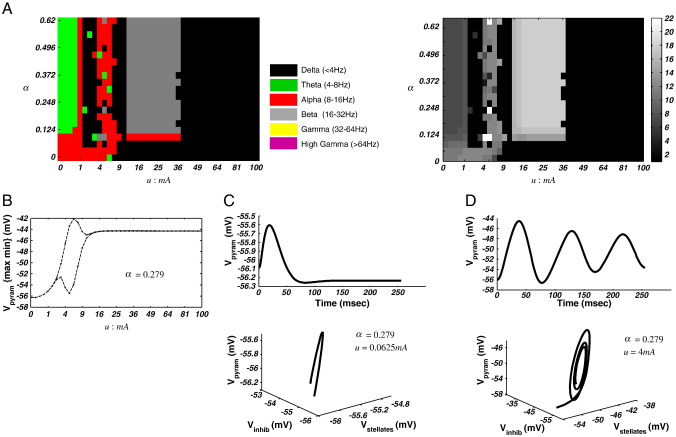
Spectral response of the neural-mass model. (A) Left: Spectral phase-diagram illustrating the maximal frequency band over the *u* and *α* dimensions. Right: phase-diagram illustrating the maximum frequency with 1 Hz resolution, which illustrates the highest maximum frequency of 22 Hz. (B) Bifurcation diagram illustrating maximum and minimum pyramidal cell membrane potential along parameter *u* for *α* = 0.279. At *u* ~ 3.06 mA the system bifurcates and enters a limit-cycle attractor. Then at *u* ~ 10.6 mA, the system undergoes a second phase transition back to a fixed-point. (C) Time domain and phase domain portraits for one region of the 2-D parameter plane (*u* = 0.0625 mA; *α* = 0.279), where the system oscillates with theta frequencies around a fixed-point attractor. (D) A second region in parameter space, here at an alpha limit cycle.

**Fig. 4:**
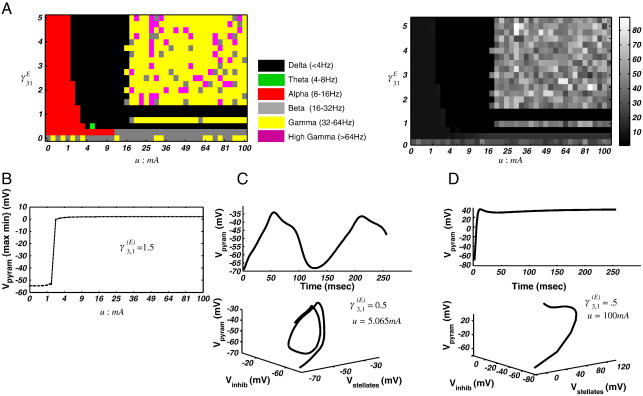
Spectral response of the mean-field model. (A) Left: Spectral phase-diagram illustrating the maximal frequency band over the *u* and *γ*_31_^*E*^ dimensions. Right: phase-diagram illustrating the maximum frequency with 1 Hz resolution. This mean-field formulation produces high frequency gamma oscillations, with the highest maximum frequency of 89 Hz. (B) Bifurcation diagram illustrating maximum and minimum pyramidal cell membrane potential along parameter *u* for *γ*_31_^*E*^ = 1.5. For this particular value of *γ*_31_^*E*^, the system remains at fixed points. (C) Time domain and phase domain portraits for a region of parameter space exhibiting a low frequency quasiperiodic attractor. (D) For high input current and high levels of forward excitatory connectivity (i.e. from the stellate cell input layer to the pyramidal cells) the system exhibits gamma resonance (*u* = 100 mA; *γ*_31_^*E*^ = 5).

**Fig. 5:**
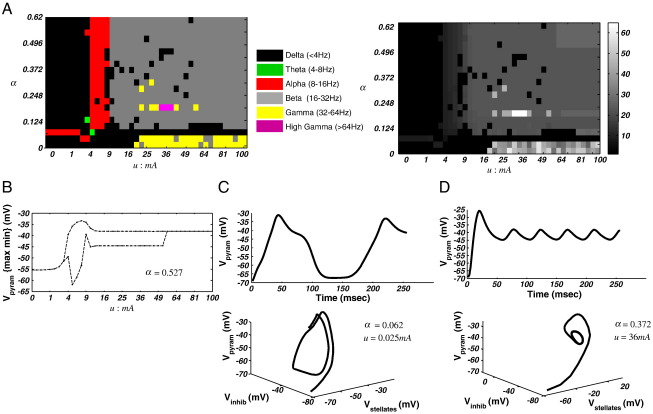
Spectral response of the mean-field model. (A) Left: Spectral phase-diagram illustrating the maximal frequency band over the *u* and *α* dimensions. Right: phase-diagram illustrating the maximum frequency with 1 Hz resolution, which illustrates the highest maximum frequency of 65 Hz. (B) Bifurcation diagram illustrating irregular bifurcation structure depending on both *u* and *α*. (C) Time domain and phase domain portraits for a region of parameter space exhibiting a low frequency quasiperiodic attractor. (*u* = 6.25 mA; *α* = 0.062). (D) A second region in parameter space, exhibiting at a beta limit cycle.

We have shown previously (Marreiros et al., 2009) that in the absence of an NMDA-like channel, the conductance-based model above exhibits resonant frequencies in the alpha and beta band, depending on the strength of the externally applied input, *u*. Here, we systematically vary two parameters and examine the spectral response at different points in parameter space (Figs. 2–5). We choose model parameters that (i) are of particular interest physiologically and (ii) have previously been shown in the ML model to induce changes from fixed-point to limit-cycle attractors. Specifically, the input current *u* (Fig. 1C) has been shown to induce bifurcations (Breakspear et al., 2003) and was included in all our searches. This input current was set to a constant value, over all times of the response. The other parameters we changed were the intrinsic excitatory connectivity*γ*_3, 1_^(*E*)^ : = *γ*_3, 1_, controlling glutamatergic input to the pyramidal cells, and the NMDA channel parameter *α*_*NMDA*_, determining kinetics at pyramidal and inhibitory subpopulations. These were chosen as exemplar parameters controlling the strength and nature (nonlinearity) of intrinsic coupling. We therefore performed two searches of bi-dimensional parameter space for pairs {*u*, *γ*_3, 1_} and {*u*, *α*} for both the NMM and MFM instantiations of the ML model. The values used for these simulations (Figs. 2–5) cover two orders of magnitude around their nominal values to ensure we considered a wide and physiological regime of parameter space. This section uses a combination of numerical techniques (integrating Eq. (5) for the mean-field treatment and Eqs. (7) and (8) for the neural-mass treatment) and linear stability analysis to illustrate the sort of dynamics these models can manifest. In the next section, we address how their dynamics can be predicted (generated) formally, in terms of spectral density.

### Neural-mass dynamics

Here, we present a descriptive bifurcation analysis of the models above. Strictly speaking, a (local) bifurcation occurs when a parameter change causes the stability of an equilibrium (or fixed point) to change. However, here we use bifurcation (and phase transition) in a slightly informal and inclusive sense to refer to any qualitative or topological change in behaviour, including those of transient responses to perturbation. Technically, this means we are interested in loss of stability (when any real eigenvalue ceases to be negative) and the emergence of oscillations (when any imaginary eigenvalue ceases to be zero). To access these changes, we characterise transient dynamics as quasiperiodic, even if the attractor is a fixed point. Given the size of the state-space we did not characterise the particular type of bifurcations encountered (analytically), but they likely belong to a set of Hopf, saddle node and hetero/homoclinic bifurcations.

We first examined the system response over the parameter space *u* × *γ*_3, 1_. To do this, all parameters were set to their nominal values (see Table 1), while *u* and *γ*_3, 1_ were varied systematically: *u*: 0 → 100 mA; *γ*_3, 1_: 0 → 5. The system of differential equations (Eq. (5) and Fig. 1C) was integrated for 256 ms and the pyramidal cell membrane potential (*V*_3_) was taken to represent the observable response or output (Moran et al., 2007). We created bifurcation diagrams from each integration step representing {max(*V*_3_), min(*V*_3_)}over 0 to 256 ms. We examined regions of parameter space where (max(*V*_3_) = min(*V*_3_)) after an initial (120 ms) transient and defined these regions as fixed points. When (max(*V*_3_) > min(*V*_3_)), we defined these regions as (pseudo) limit-cycle regimes. Clearly, these dynamics could just represent protected transients that may or may not have a true limit cycle. However, they indicate the emergence of at least one imaginary eigenvalue.

Having established the type of dynamics (fixed-point or quasiperiodic), we summarised the corresponding frequency domain output, *g*(*ω*). For regions of parameter space where the system settled to a fixed-point, we assumed that physiological fluctuations in *u*(*t*) cause the observed spectral output. The implicit filtering of these fluctuations is encoded in the system's modulation transfer function. The transfer function *H*(*jω*) was computed for radial frequencies *ω* corresponding to 0 to 100 Hz, using standard linear systems theory, where the requisite derivatives were evaluated numerically (as described in Moran et al., 2009). The fixed point was taken as the numerical solution of the mean-field states *μ*(*t*) at 240 ms.

(9) $$\begin{array}{c} H(j\omega ,\mu )=\frac{\partial h}{\partial \mu }{\left(Ij\omega -\frac{\partial f}{\partial \mu }\right)}^{-1}\frac{\partial f}{\partial u}\\ g(\omega )=|H(j\omega ,\mu ){|}^{2}\phi (\omega )\\ h(\mu )=\mu_{V_{3}} \end{array}$$

Here, **h**(*μ*) = *μ*_*V*_3__ maps hidden states to observed measurements and *φ*(*ω*) corresponds to the spectral density of the fluctuations in *u*(*t*), which, for simplicity, we assumed were white; i.e., *φ*(*ω*) = 1. It is important to realise that these perturbations are not the random fluctuations *Γ* on the states of neurons in the population but perturbations of a mean-field sort that are seen identically by all neurons. The spectral density *φ*(*ω*) of these fluctuations is generally unknown and, in the context of DCM, has to be estimated.

For regions of parameter space that generated limit cycles (or quasiperiodic transients), we assumed the spectral activity would be dominated by the deterministic dynamics of the ensemble mean and computed the characteristic frequency from the numerical solution (orbit) directly, over 256 ms (the length of each integration or solution from the initial conditions). These orbits included any quasiperiodic or chaotic transients after perturbation. The spectra were obtained using a parametric autoregressive (AR) model of order *p* where

(10) $$\begin{array}{l} V_{3}(t)=\sum_{i=1}^{p}a_{i}V_{3}(t-i)+\varepsilon (t)\\ V_{3}=h(\mu )=\mu_{V_{3}} \end{array}$$

The autoregressive coefficients *a*_*i*_ : *i* = 1, …, *p* provide a direct estimate of the spectral density *g*(*ω*), using the following transform:

(11) $$H{\left(j\omega \right)}^{-1}=\sum_{i=1}^{p}a_{i}e^{\mathrm{ij\omega}}$$

The estimation of the autoregression coefficients used the spectral toolbox in SPM (http://www.fil.ion.ucl.ac.uk). Details can be found in Roberts and Penny (2002). Note that we could have used the transfer function approach (Eq. (9)) for both steady-state and limit-cycle regimes. However, this would have given a limited, ‘snapshot’ of the spectrum at one point on the limit cycle. Later, we will use the transfer function averaged over the limit cycle to predict spectral responses; however, here we can just use the numerical (AR) estimate (Eq. (10)) to characterise simulated dynamics.

Using the peak of *g*(*ω*)for each {*u*, *γ*_3, 1_} pair, we identified the regions of parameter space that generated predominant delta (< 4 Hz), theta (> 4–8 Hz), alpha (> 8–16 Hz), beta (> 16–32 Hz), gamma (> 32–60 Hz) or high-gamma (> 60 Hz) oscillations (Fig. 2A). The ensuing bifurcation diagram showed that the system bifurcates at a particular value of *γ*_3, 1_ (*γ*_3, 1_ = 0.5), when all other parameters are fixed at their nominal value as per Table 1. In Fig. 2B, we plot the {*max min*} system response as a function of *u*. We observe that for values of *u <* 3.06 mA the system exhibits a stable equilibrium point, where the modulation transfer function is dominated by low frequency delta activity and a prominent alpha response for low values of *γ*_3, 1_ (Fig. 2C). Then at *u* ~ 3.06 mA the system bifurcates to a quasiperiodic attractor with periodicity in the alpha range. In Fig. 2D, we illustrate the time domain response of the pyramidal cell membrane potential and the systems phase-space in three dimensions {*V*_1_, *V*_2_, *V*_3_} in this regime. The system later settles to a new equilibrium at *u* > 10.6 mA with a narrow beta resonance for *γ*_3, 1_ = 0.5 (Fig. 2E).

Repeating the procedure for parameter pair {*u*: 0 →100 mA; *α*_*NMDA*_: 0 →0.62}, we obtain a different phase-diagram (Fig. 3A). In this case, the bifurcation structure is largely dependent on *u*. For *α*_NMDA_*=* 0 the system remains in equilibrium for all values of *u*. Then at values of *α*_NMDA_ > 0, and *u >* 3.06 mA, a phase transition produces a limit cycle. As above, a return to a fixed point is observed after a second phase transition at *u* ~ 10.6 mA. Examining the spectral peak we observe, for low values of *α*_NMDA_ and *u*, an alpha regime mediated by the modulation transfer function. Increasing *α*_NMDA_ leads to a decrease in the MTF's spectral peak to theta frequencies (Fig. 3C). Similarly, in the (transient) quasiperiodic regime, the system changes to produce delta, theta, alpha, or beta peaks, depending on both *u* and *α*_NMDA_ (Fig. 3A). Then, after the final phase transition, a fixed-point with a beta MTF first emerges, followed by a new delta equilibrium for *u* > 36 mA.

### Mean-field dynamics

The system response for the full mean-field formulation was assessed in the same way. This formulation contains new states that represent the dynamics of the population covariance, leading to spectral outputs that differ from those of the NMM. We obtained the maximum and minimum system output, *V*_3_, over bi-dimensional parameter searches as above, characterising the attractor at each parameter pair as either a fixed point {max(*V*_3_) = min(*V*_3_)} or quasiperiodic {max(*V*_3_) > min(*V*_3_)}. Then using the modulation transfer function at 240 ms or AR estimates over the full 256 ms, we evaluated the spectral response. First, we focused on the parameter pair {*u*: 0 → 100 mA; *γ*_3, 1_: 0 → 5} to examine the effects of varying the strength of the input current and intrinsic excitatory connectivity. At *γ*_3, 1_= 0, we found a fixed-point attractor with beta/gamma resonance for all *u* i.e. where (max(*V*_3_) = min(*V*_3_)) after 120 ms (Fig. 4A). The system then undergoes a series of bifurcations along both the *u* and *γ*_3, 1_ dimensions. For 0.25 < *γ*_3, 1_ ≤ 0.5 the system exhibited beta and low frequency delta limit-cycle oscillations for values of *u* > 4 mA (Fig. 4A and C) and alpha resonance for *u* < 4 mA. Then for *γ*_3, 1_< 1, the system returns to a fixed-point attractor, with alpha, delta, beta and gamma resonances, depending on *u.* At *γ*_3, 1_ = 1, the system bifurcates to a new low frequency limit cycle for *u* > 7.56 mA. For *γ*_3, 1_= 1.25 a similar bifurcation occurs but here, only for values of *u* = 3.06 mA. Finally for all *γ*_3, 1_≥ 1.5, the system has fixed-point attractors, with resonance frequencies dependent on *u* (Fig. 4B and D).

The second bifurcation analysis of the mean-field model looked at the parameter pair {*u*: 0 → 100 mA; *α*_*NMDA*_: 0 → 0.62}. The spectral response pattern is given in Fig. 5A. As with the previous search, the MFM displays a bifurcation diagram that depends on both model parameters. When *0* < *α*_NMDA_*<* 0.031 the system has a fixed-point attractor with delta, beta and gamma resonances, depending on *u* (Fig. 5A). Then at values of *α*_NMDA_ = 0.062, the system produces low frequency limit cycles for values of *u* > 3.0625 mA (Fig. 5C). For *α*_NMDA_> 0.062 the system displays limit cycles again, for values of *u* > 3.062 at beta, gamma and high-gamma frequencies (Fig. 5D); however, these limit-cycle dynamics undergo a second bifurcation back to fixed points at certain *α*_NMDA_ values (Fig. 5B). Second transitions occur close to 60.06 mA for values of *α*_NMDA_ = {0.093, 0.0186 and 0.527 → 0.62}.

### Diffusion on a limit cycle

In the analysis above, we used parameters encoding different synaptic processes to generate a landscape of spectral responses (bifurcation and phase-diagrams). These parameters affect the flow of the deterministic dynamics in Eq. (5). Another parameter that is of particular interest in the setting of mean-field limit cycles is the amplitude of stochastic terms that determines the diffusion operator *D* (Table 1).

Gang et al. (1993) have shown that diffusion can affect several properties of deterministic oscillatory responses. In a model system comprising two states, they showed that in the region of a saddle node bifurcation, diffusion can change the peak frequency and spectral height of the response in a nonlinear way. Since the Morris–Lecar model has a similar bifurcation structure (Tateno and Pakdaman, 2004; Rinzel and Ermentrout, 1998), we examined the effects of diffusion close to transitions from fixed-point to periodic attractors. Our high dimensional, coupled system, though not amenable to analytical solution, does display some phase transitions that could resemble heteroclinic (a trajectory joining one equilibrium to another) or homoclinic (a trajectory joining a saddle point to itself) orbits, namely, low frequency oscillations and asymmetric phase portraits (Rinzel and Ermentrout, 1998; Fujii and Tsuda, 2004); see Figs. 4C and 5C.

**Fig. 6:**
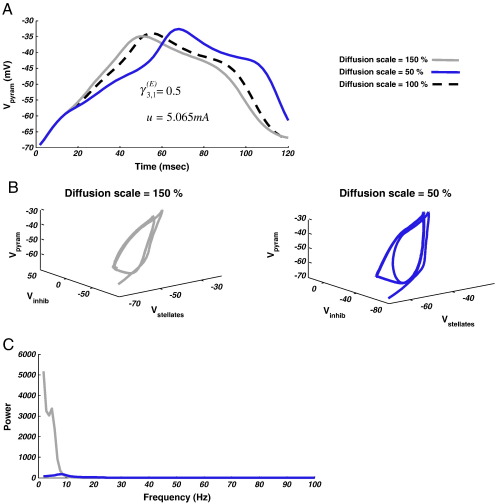
Diffusion on a limit cycle. (A) The mean-field model as shown in Fig. 4C (*u* = 5.065 mA; *γ*_31_^*E*^ = 0.5), exhibits low frequency, theta limit-cycle oscillations (dashed line). Adding state noise to the system by increasing the diffusion coefficient causes the orbit to speed up at *V*_pyram_ ~ -40 mV, while decreasing the strength of the diffusion coefficient slow it down. (B) Phase domain plots showing greater symmetry (resp. asymmetry) in the orbit at D = 150% (resp. 50%) baseline. (C) Frequency spectra show a higher peak frequency for D = 150%.

To investigate the effects of diffusion, we simulated the system at {*γ*_3, 1_= 0.5; *u* = 5.065 mA} and scaled *D* to 150% and 50% of its nominal value (Table 1). For a diffusion scaling of 150% we observed, in the time domain, a faster response (grey line, Fig. 6A), close to the asymmetry at *V*_*3*_ = -40 mV, than the original output (hashed line, Fig. 6A). For a diffusion scaling of 50%, however, we observed an enhanced slowing at this point (Fig. 6A, blue line), i.e. *V*_*3*_ flattens even further*,* as per Gang et al. (1993). We can also see how increased diffusion (system or state noise) adds symmetry to the periodic orbit (Fig. 6B). The result in the frequency domain is a decrease in power for low diffusion dynamics compared to the increased diffusion (Fig. 6C). This paradoxical speeding with increased diffusion may reflect an increased diffusion away from the heteroclinic or homoclinic orbit (Gang et al., 1993).

In summary, the Morris–Lecar model, with NMDA currents, can display a rich repertoire of dynamical behaviours. These behaviours, under steady-state or ergodic assumptions, can be accessed through conventional linear systems analysis (via the modulation transfer function) but also arise from bifurcations due to changing key parameters of the system's stochastic differential equations. These dynamics are seen even in simple neural-mass approximations to the ensemble activity but are more evident under a mean-field treatment that preserves dynamics in the dispersion of hidden states by random fluctuations. The presence of bifurcations means that any generative model based on these neuronal models has to accommodate the implicit phase transitions. We now address this issue:

## Consistent spectral prediction

To invert models based on the systems described in the previous section (given real data) we require a generative model of their spectral behaviour. In the simulations above, we used two procedures to estimate the spectral output, depending on the type of attractor network. At fixed points, the modulation transfer function was used to compute the spectrum, assuming small perturbations by noisy input. In contrast, in the case of quasiperiodic transients and attractors, we used an AR model to compute the spectrum from a numerical solution or orbit. In this section, we outline a new approach that provides a generic approximation to the spectral response for systems with any sort of attractor. The approach is motivated by two converging ideas in dynamical systems theory: the principle of dynamic enslavement (Haken, 1977) that arises from the centre manifold theorem (Guckenheimer and Holmes, 1983), and the characterisation of noisy precursors to nonlinear instabilities (Wiesenfeld, 1985; Knobloch and Wiesenfeld, 1983).

The centre manifold theorem is used to simplify the dynamics of a nonlinear system at or near a bifurcation point. At a bifurcation point, a small number of system modes (mixtures or patterns of states) lose stability, such that these modes exhibit slow, large oscillations, while the remaining modes decay quickly. Hence it is possible to approximate the dynamics using only the motion of slow modes on a centre manifold (Knobloch and Wiesenfeld, 1983). This can be done by breaking the system into two blocks (a slow Jordan block, *A* and a fast Jordan block, *B*). The stable or fast dynamics of modes (with large negative real eigenvalues) become enslaved by the unstable or slow modes (with small or zero real eigenvalues). Mathematically, the dynamics of some states *z*(*t*) can be expressed as

(12) $$\dot{z}=F(z,u)$$

These can be decomposed into coupled differential equations describing the motion of slow *μ*(*t*) and fast *η*(*t*) modes respectively (Carr, 1983).

(13) $$\begin{array}{c} \dot{\mu }=A\mu +\varepsilon (\mu ,\eta ,u)\\ \dot{\eta }=B\eta +g(\mu ,\eta ,u)\\ z=\{\mu ,\eta \} \end{array}$$

Here, we associate the slow stable modes with the sufficient statistics describing population activity above, *μ* = {*μ*^(1)^, …, *Σ*^(1)^, …}. Crucially, Eq. (13) shows that the motion of these modes can be expressed in terms of a slow (linear) part and fast fluctuations due to exogenous input (and any un-modelled fast modes). This means we can predict the spectral response to fast fluctuations from the (slow) Jordan block *A* in Eq. (13). This corresponds to the Jacobian *A* = ∂ **f**/∂ *μ* of Eq. (5), when evaluated on, and only on, the centre manifold. The Jacobian specifies the transfer function, which specifies the spectral responses to fast fluctuations. Under ergodic assumptions, this means the expected transfer function can be evaluated by taking its average over a solution or orbit of the system; because the orbit must be on (or near) the centre manifold. Using linear systems theory, this gives the following predictor of spectral activity, where the spectral density of the fluctuations is *ϕ*(*ω*)

(14) $$\begin{array}{l} H(j\omega ,\mu )=\frac{\partial h}{\partial \mu }{\left(Ij\omega -\frac{\partial f}{\partial \mu }\right)}^{-1}\frac{\partial f}{\partial u}\\ g(\omega )={\langle \left|H(j\omega ,\mu ){|}^{2}\phi (\omega \right)\rangle }_{\mu \in \mu (t_{1}),\ldots ,\mu (t_{T})} \end{array}$$

Formally, this is exactly the same as Eq. (9) but averaging over the orbit (solution) of the system by sampling at *t*_1_, …, *t*_*T*_, as opposed to just one time point. By sampling phase-space selectively in this way, we construct a spectral profile that reflects macroscopic dynamics on the centre manifold that enslave transverse fluctuations.

**Fig. 7:**
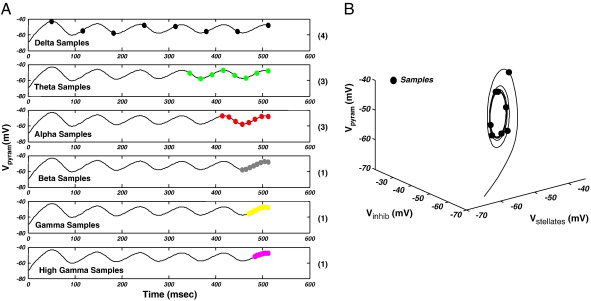
Sampling phase-space. (A) Six possible sampling sets for an orbit. Samples are distributed for delta, theta, alpha, beta, gamma or high gamma (colour coded as per Figs. 2–5). The time series is differentiated at the sample points in each set. In parentheses, we show the number of derivatives greater than zero. The set with greatest sign balance (i.e. number positive derivative = 4) is chosen as the sampling set. (B) Alpha limit cycle of NMM as per Fig. 2C, sampled using the set algorithm.

To sample an orbit, we use discrete sampling points, spread evenly over the orbit. To optimise this set of points we test six sampling periods (corresponding to one cycle of a delta, theta, alpha, beta, gamma and high-gamma oscillation). We then use the sample set with the best balance of positive and negative derivatives. This is because points spread evenly over the orbit should encounter the same number of increases and decreases. This sampling is somewhat arbitrary but works well in practice. Fig. 7A, illustrates this by showing that an alpha limit cycle (NMM; *γ*_3, 1_= 0.5; *u =* 4 mA) sampled according to this scheme has balanced derivatives at alpha, theta and delta sampling frequencies. Fig. 7B illustrates the procedure in phase-space.

The nice thing about this approximation is that it gives the same frequencies as the (numerical AR-based) frequency analysis of the orbits used to characterise the spectral activity of quasiperiodic dynamics. This means the same (consistent) approximation can be used for fixed-point attractors and quasiperiodic attractors (or indeed any attractor). Crucially, this removes qualitative differences between the spectral activity before and after a phase transition: One can see this anecdotally by noting that the frequency profile of the dynamics will be dominated by the principal mode (with the largest eigenvalue of *A* = ∂ **f**/∂ *μ*). As the system undergoes a bifurcation, the real part of the eigenvalue crosses (reaches) zero; however, the frequency will not change markedly, because it is determined by the imaginary part. Another perspective on the approximation in Eq. (14) is that we are estimating the linearised frequency response to perturbations from the system's orbit. The frequencies of the fast (stable) modes will contribute much less than those of the slow (unstable) modes, because they dissipate much more quickly; where the dissipation of the perturbation to each mode is proportional to the mode's (negative) real eigenvalue.

This procedure for predicting or generating a spectral output is clearly valid at fixed points, where the Jacobian is necessarily constant. In order to motivate the same procedure for estimating spectra from quasiperiodic attractors, we appeal to the work of Knobloch and Wiesenfeld (Wiesenfeld, 1985; Knobloch and Wiesenfeld, 1983). Their work describes the characteristics of a dynamical system on a quasiperiodic attractor when it is subject to noise. Specifically, it illustrates how small perturbations kick the system off the limit cycle and how it then relaxes back to a periodic orbit. These transients are governed by the transfer function at the point of perturbation and contribute more to the observed output as the system approaches instability (slows down). We assume that these perturbations or ‘kicks’ are elicited here by exogenous fluctuations (e.g., afferent neuronal activity) or un-modelled fast modes intrinsic to each region.

**Fig. 8:**
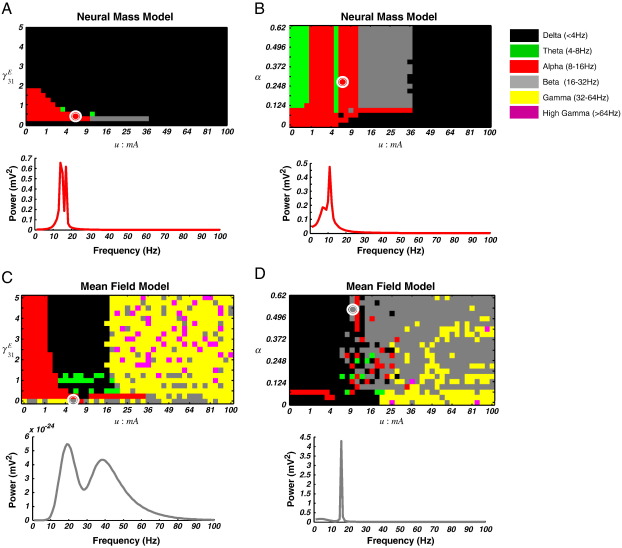
Sampled spectral responses. (A) Top: Spectral phase-diagram illustrating the maximal frequency band over the *u* and *γ*_31_^*E*^ dimensions for the neural-mass model using the modulation transfer function sampled from the centre manifold. The diagram shows a good comparison between this and the full (non-sampled) estimates shown in Fig. 2A. Bottom: The spectral prediction at *u =* 6.25 mA and *γ*_31_^*E*^ = 0.25 (white circle) contains a peak at 14 Hz, in the alpha range and a secondary peak at 17 Hz, in the beta range. This illustrates the ability of the model to generate multimodal spectra. (B) Top: Spectral phase-diagram illustrating the maximal frequency band over the *u* and *α* dimensions for the neural-mass model. The diagram shows smoother transitions than in Fig. 3A. Bottom: The spectral prediction at *u =* 5.06 mA and *α* = 0.279 (white circle) contains peaks in the alpha (11 Hz) and theta (7 Hz) range. (C) Top: Spectral phase-diagram illustrating the maximal frequency band over the *u* and *γ*_31_^*E*^ dimensions for the mean-field model. These responses have a similar profile to Fig. 4A. Bottom: For the MFM, the sampling scheme also produces multimodal spectra, with beta (19 Hz) and gamma (39 Hz) peaks for *u =* 5.06 mA and *γ*_31_^*E*^ = 0 (white circle). (D) Top: Spectral phase-diagram illustrating the maximal frequency band over the *u* and *α* dimensions for the mean-field model. These responses have a similar profile to Fig. 5A. Bottom: For *u =* 9 mA and *α* = 0.56 (white circle), the prediction contains two peaks, one in the beta range at 16 Hz, and one in the theta range at 4 Hz.

Using this sampling procedure, we recreated the spectral phase-diagrams, illustrating the maximal frequency band over the parameter pairs (Fig. 8). From these graphs we can see that the sampled attractors are in good agreement with the AR and (fixed point) MTF spectra. Importantly, the model can also generate multimodal spectra in various regions of parameter space. This suggests that the sampling method is sensitive to a range of frequencies; please see Fig. 8. For the neural-mass model in the {*u*, *γ*_3, 1_} plane we observe similar alpha-theta-beta transitions to delta activity, while for this model in the {*u*, *α*} plane, smoother transitions from theta fixed points to alpha limit cycles to beta fixed points are obtained than in the non-sampled case (Fig. 8A and B). For the mean-field model, both pairs retain their high frequency attractors, with beta, gamma and high-gamma speckled in the high-valued regions of parameter space.

In summary, this section has introduced a simple and internally consistent way of generating the spectral responses of any dynamical system subject to random perturbations. Crucially, these responses can be generated in the same way, irrespective of whether the system exhibits fixed points, quasiperiodic transients or chaotic attractors. Furthermore, this spectral characterisation will not (in general) show discontinuities at bifurcations or phase transitions. This is important from the point of view of model inversion, as we will see in the next section.

## Simulations

In this section, we highlight the importance of using a consistent generative model of spectral responses when inverting a DCM. Model inversion means estimating the conditional density of the unknown model parameters *p*(*ϑ*|*g*(*ω*), *m*) given observed spectral density features **g**(*ω*) (predicted by *g*(*ω*, *ϑ*)) for any model *m*. Each model is defined by the network architecture and priors on the parameters, *p*(*ϑ*|*m*). These unknown parameters include the biophysical parameters of the neural-mass or mean-field model, as well as parameters controlling neuronal and measurement noise (Table 1: see Moran et al., 2009 for details). The model is inverted using standard variational approaches described in previous publications (Stephan et al., 2008; David et al., 2005) and summarised in Friston et al. (2007). These procedures use a variational scheme in which the conditional density is optimised under a fixed-form (Laplace) assumption. This optimisation entails maximising a free-energy bound on the log-evidence, ln *p*(**g**|*m*). Once optimised, this bound can be used as an approximate log-evidence for model comparison (Stephan et al., 2009; Penny et al., 2004). Priors on the parameters play an important role in this scheme. Firstly, for a given model, the priors are an important determinant of the log-evidence. This can be seen by expressing the log-evidence in terms of accuracy and complexity (Friston et al., 2007), where complexity reflects the discrepancy between the prior and posterior densities. For example, when posterior estimates are required to diverge greatly from the priors to explain the data, a larger penalty is accrued, compared to posterior parameter estimates that are similar to their priors. Second, in our implementation of DCM, priors are used to initialise the parameter estimates. This has important implications for the optimisation scheme, which performs a gradient ascent on the free-energy (or approximate log-evidence). We will use the phase-diagrams in Figs. 2–5A to specify priors (and implicitly initialisation of the inversion scheme).

**Fig. 9:**
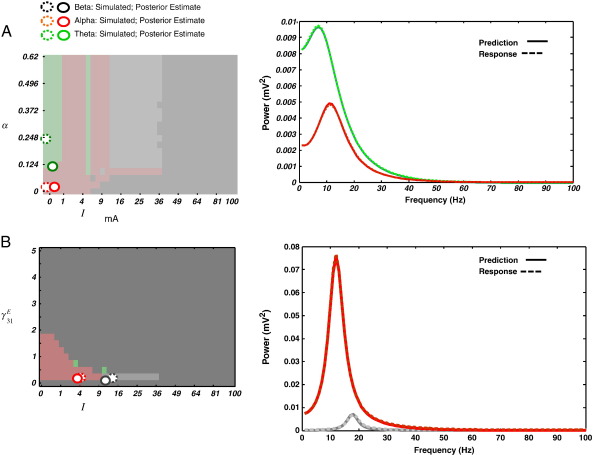
Simulations. (A) Two trials with different spectral profiles generated from the NMM. At (*u* = 0. 25 mA; *α* = 0.031) we generated an “alpha trial” from fixed-point resonance (hashed red circle & spectrum) and at (*u* = 0. 25 mA; *α* = 0.248) we generated a “theta trial” from fixed-point resonance (hashed green circle and spectrum). Model fits and MAP estimates are shown as full circles in the phase-diagram (reproduced from Fig. 3) and full lines in the spectra plots. (B) Two trials with different spectral profiles generated from the NMM. At (*u* = 4 mA; *γ*_31_^*E*^ = 0.25) we generated an “alpha trial” from an alpha limit cycle (hashed red circle and spectrum) and at (*u* = 14.063 mA; *γ*_31_^*E*^ = 0.25), we generated a “beta trial” from fixed-point resonance (hashed grey circle and spectrum). All other parameters are as per Table 1. Model fits and MAP estimates are shown as full circles in the phase-diagram (from Fig. 2) and full lines in the spectra plots. The colours of the lines in the plots correspond to the different trials coloured according to their predominant frequency.

Our simulations try to demonstrate the usefulness of the consistent spectral predictor in Eq. (14), when dealing with phase transitions during model inversion. We generated two sampled spectra from models with different predominant frequencies and inverted both as if they belonged to two trials (or trial averages) in an experimental data set. First, we illustrate model inversion using spectra generated by networks with fixed points (i.e., no phase transition but a difference in the principal frequency, under small perturbations). For this we simulated spectra at an alpha fixed-point (Fig. 3A), using *u =* 0.06 mA and *α*_NMDA_= 0.03 (trial 1) and a theta fixed-point, using *u =* 0.06 mA and *α*_NMDA_= 0.25 (trial 2)*,* with all other parameters as per Table 1. The priors on these parameters were set to *u =* 1 mA and *α*_NMDA_= 0.06*,* with all other parameters set to their nominal values (Table 1). Fig. 9A illustrates the *maximum a posteriori* (MAP) estimates for this inversion and the corresponding model fits. MAP estimates for trial 1 placed *u =* 0.6 ± 0.22 mA (posterior mean ± s.d.) and *α*_NMDA_= 0.03 ± 0.01. The parameters for trial 2 were estimated in the direction of the simulated change in the NMDA channel function, with estimates *u =* 0.76 ± 0.27 mA and *α*_NMDA_= 0.10 ± 0.02, resulting in non-overlapping 95% posterior confidence intervals for *α*_NMDA_(trial 1) and *α*_NMDA_(trial 2). Though we found a change in the correct direction for *α*_NMDA_ in trial 2 relative to trial 1, the posterior mean for trial 2 did not reach its simulated value of 0.25. We looked for possible identifiability issues by examining the posterior correlations among parameters. The maximum (absolute) correlation was with the parameter encoding the fixed variance (diffusion) measure, which correlated negatively with *α*_NMDA_ (*ρ* = − 0.4) and may account for the small values of *α*_NMDA_ estimates in trial 2.

For our second analysis, we generated spectral data from both sides of a phase transition (Fig. 2A and B). We generated an alpha limit cycle at *u =* 4.00 mA and*γ*_3, 1_ = 0.25 (trial 1) and a beta spectrum after a phase transition to a fixed-point, at *u =* 14.06 mA and *γ*_3, 1_ = 0.25 (trial 2). Priors on these parameters were set to *u =* 0.06 mA and*γ*_3, 1_ = 0.13*,* with all other parameters set to their nominal values (Table 1). Fig. 9B illustrates the *maximum a posteriori* (MAP) estimates for this inversion and the corresponding model fits. MAP estimates for trial 1 placed *u =* 3.65 ± 0.28 mA and *γ*_3, 1_= 0.22 ± 0.14. Crucially, trial 2 estimates crossed the phase transition with estimates of *u =* 11.79 ± 0.00 mA and *γ*_3, 1_= 0.11 ± 0.04.

In summary, these results show that the inversion scheme, which requires a continuous free-energy function, can accommodate bifurcations (in this example, at *u* ~ 10.6 mA). The reason for this is that the spectral behaviour of the system does not change qualitatively with a dynamical phase transitions. We have exploited this by using spectral data features to provide a free-energy objective function for model inversion that is analytic (smooth and continuously differentiable).

## Discussion

Converging evidence suggests that oscillations in cortical dynamics play an important role in mediating and coordinating activity in neural circuits (Womelsdorf and Fries, 2007; Sejnowski and Paulsen, 2006; Seidenbecher et al., 2003; Dragoi and Buzsáki, 2006). Biophysically plausible generative models of these oscillations allow us to infer the synaptic physiology that subtends observed responses. In this context, we addressed two aspects of biological realism when constructing our generative model.

First, we attempted to capture some important physiological processes using a greater array of channel types than considered previously (Moran et al., 2009). The inclusion of NMDA channels is important in DCM hypotheses testing (i.e., model comparison) and inference, given the potential role of NMDA receptors in a wide range of cognitive processes (e.g. memory; Nakazawa et al., 2004, and learning; Chen and Tonegawa, 1997) and pathophysiology (e.g. in schizophrenia, Stephan et al., 2009). Establishing the utility of the modelling scheme, given real data, will require a series of careful validation studies. This entails the study of electromagnetic signals when certain channel properties are changed selectively or measured independently; e.g. via microdialysis (Moran et al., 2008) or pharmacological manipulations. This is the subject of current work, in which we focus on NMDA receptor function and, crucially, interactions with things like dopaminergic neurotransmission. In this context, we envisage that questions about the form of the model (e.g., the need to include NMDA receptors) will be addressed using Bayesian model comparison, in other words, comparing the evidence for models with and without NMDA receptors.

Second, the repertoire of our generative models has been extended to provide for nonlinear oscillatory behaviour based on limit-cycle attractor networks, a ubiquitous property of complex systems (Jirsa, 2004) and density dynamics that conform to nonlinear Fokker–Planck equations (Breakspear et al., 2010).

Studies investigating the bifurcation structure of Morris–Lecar (ML) models have indicated the existence of distinct phase transitions in one- and two-dimensional parameter spaces. One-dimensional analysis has revealed two different types of oscillatory solutions; the first corresponds to saddle node bifurcations, which produce slow oscillations, with frequencies proportional to the applied driving current. In the second, a higher current leads to stable limit cycles following a sub-critical Hopf bifurcation (Ermentrout, 1996; Rinzel and Ermentrout, 1998; Tateno and Pakdaman, 2004). In two-dimensional parameter space, transitions between these solutions can be achieved through global homoclinic bifurcations by altering particular currents within the model (Tsumoto et al., 2006). Clearly, the bifurcation structure of the ML model is rich and complex. Our primary concern in this paper was not to characterise the bifurcations per se, but instead to search a wide parameter space, within a meaningful physiological range, to elucidate the spectral response profile. We did this by applying a numerical bifurcation analysis to study the stationary regimes of the ensemble model, which covered spectral responses from low, delta frequencies (2 Hz) to high gamma (100 Hz). We found that our NMM and MFM formulations generated distinct spectra in the associated phase-diagrams, with higher frequency gamma responses observed for the MFM. On the other hand, lower frequency oscillations could be generated by both. Low frequency dynamics at fixed points suggest that both of these formulations might act as generative models for other forms of DCM, e.g. DCM for ERPs (Kiebel et al., 2006).

The proposed approach to spectral prediction (generation) allows us to observe spectra consistently from all regions in parameter space; at fixed points, at bifurcation to quasiperiodic behaviour and beyond. The ideas from Wiesenfeld (1985) allow the latter two regions to be treated similarly. Here the dimension reduction at the centre manifold was extended to limit-cycle bifurcations and specifically its noisy precursor. Perturbations from the limit-cycle attractor (centre manifold) can be approximated with linearised transients. At the fixed-point bifurcations the sampling procedure embodies an adiabatic reduction, or the enslaving principle introduced by Haken (1977). The Jacobian at a fixed-point may be an over-complete representation of the deterministic spectral response, but allows for a consistent generative model of oscillatory dynamics under the assumption of stochastic perturbations.

In this paper, we simulated spectral responses using both the MFM and NMM models of one cortical region. In subsequent work we will extend this approach to multiple brain regions. The case of multiple interacting cortical sources is mathematically identical to the treatment of a single source in this paper. This is because, for multiple regions, we simply augment the coupling parameters *γ*_*i*, *j*_^*k*^ in Eq. (6) to include (extrinsic) coupling between regions, where the strength of AMPA mediated input would correspond to the strength of extrinsic glutamatergic connections. One might anticipate that quasiperiodic dynamics will be more prescient in this context because heterogeneity in (extrinsic and intrinsic) coupling may induce more complex macroscopic dynamics (see Shibata and Kaneko, 1997; Hennig and Schimansky-Geier, 2008; Stefanescu and Jirsa, 2008 and De Monte et al., 2003). For such inter-regional connections the presynaptic population is the pyramidal cell population, while the postsynaptic population can be any of the three neuronal types (pyramidal, spiny stellate or inhibitory interneurons), depending on the type of connection (forward, backward, lateral).

In conclusion, this paper makes two contributions to the ongoing development of dynamic causal models of electrophysiological data: it augments recent conductance-based models with an NMDA channel, and it introduces a generic and consistent method for generating spectral responses across different regions of parameter space. This might be an important step for underwriting the robustness of model inversion in DCM, when analysing nonlinear systems with phase transitions.

## Figures and Tables

**Table 1:** Priors for model parameters of the observation model and neuronal sources. These parameters operate as log-normal scaling parameters on prior means (see Moran et al., 2009 for more details).

Parameter	Interpretation	Prior	
*ϑ*_*i*_ = *π*_*i*_ exp(*Θ*_*i*_)	Mean:	Variance:
*π*_*i*_	*Θ*_*i*_ = *N*(0, *σ*_*i*_)
*Neuronal source*			
*κ*_*e*/*i*/NMDA_	Rate constants	$\begin{array}{l} \pi_{\kappa_{e}}=4{\text{ms}}^{-1}\\ \pi_{\kappa_{i}}=16{\text{ms}}^{-1}\\ \pi_{\kappa_{i}}=100{\text{ms}}^{-1} \end{array}$	$\begin{array}{l} \sigma_{K_{e}}=1/32\\ \sigma_{K_{i}}=0\\ \sigma_{K\text{NMDA}}=0 \end{array}$
*α*_NMDA_	Magnesium block	*π*_*α*_ = 0.062	*σ*_*α*_ = 1/4
*V*_*R*_	Threshold potential	*V*_*R*_ = − 40mV	*σ*_*V*_*R*__ = 0
*V*_*E,I,L*_	Reversal potentials		*σ*_*V*_*E*__ = 0
		*V*_*E*_ = 60mV	*σ*_*V*_*I*__ = 0
		*V*_*I*_ = − 90mV	*σ*_*V*_*L*__ = 0
		*V*_*L*_ = − 70mV	
*g*_*L*_	Leak conductance	*g*_*L*_ = 1	*σ*_*g*_*L*__ = 0
*C*	Membrane capacitance	*π*_*C*_ = 8/1000	*σ*_*C*_ = 0
*D*	Diffusion	*π*_*D*_ = 1/16	*σ*_*D*_ = 1/64

*Design*			
*β*	Trial specific changes	*π*_*β*_ = 1	*σ*_*β*_ = 1/8

*Observation model*			
*α*_*s*_	Channel white noise	*π*_*α*_*s*__ = 10^− 5^	*σ*_*α*_*s*__ = 1/2
*α*_*c*_	Channel pink noise	*π*_*α*_*c*__ = 10^− 5^	*σ*_*α*_*c*__ = 1/2
*L*	Lead-field gain	*π*_*L*_ = 1	*σ*_*L*_ = 1

## Appendix A

The dynamics of the density *q*(*x*), under the Fokker–Planck equation is given by

$$\dot{q}=-\nabla \cdot fq+\nabla \cdot D\nabla q$$

With flow *f* and dispersion *D*. Assuming a Gaussian form for the population densities N(μ,∑), the dynamics of the first order moments are given by

$${\dot{\mu }}_{i}=\int -x\partial_{x}(fq)dx+\int x\partial_{x}D\partial_{x}qdx$$

Where, by parts

$$\begin{array}{l} {\dot{\mu }}_{i}=-{\left[xf(x)q(x)\right]}_{-\infty }^{\infty }+{\int f(x)q(x)dx+[xD\partial_{x}q]}_{-\infty }^{\infty }-\int D\partial_{x}qdx\\ {\dot{\mu }}_{i}=\int f(x)q(x)dx \end{array}$$

noting *q*(*x*), ∂ _*x*_*q*(*x*) → 0 as *x* → ± *∞*. And for the second order moments, where ${\bar{x}}_{i}=(x_{i}-\mu_{i})$, by parts again

$${\dot{\Sigma }}_{\mathrm{ij}}=\int ({\bar{x}}_{j}f_{i}(x)+{\bar{x}}_{i}f_{i}(x))q(x)dx+D_{\mathrm{ij}}+D_{\mathrm{ji}}$$

Under Gaussian assumptions ${\langle {\bar{x}}_{i}\rangle }_{q}=0$ and ${\langle {\bar{x}}_{i}{\bar{x}}_{j}\rangle }_{q}=\Sigma_{\mathrm{ij}}$, giving finally (main text)

$$\begin{array}{l} {\dot{\mu }}_{i}^{\left(j\right)}=f_{i}^{\left(j\right)}(\mu ,\sum ,u)+\frac{1}{2}tr(\sum^{\left(j\right)}\partial_{\mathrm{xx}}f_{i}{}^{\left(j\right)})\\ {\dot{\sum }}^{\left(j\right)}=\partial_{x}f^{\left(j\right)}\sum^{\left(j\right)}+\sum^{\left(j\right)}\partial_{x}f^{(j)T}+D^{\left(j\right)}+D^{(j)T} \end{array}$$

Please see Marreiros et al. (2009) for further details.
